# Proposal and validation of a method to classify genetic subtypes of diffuse large B cell lymphoma

**DOI:** 10.1038/s41598-020-80376-0

**Published:** 2021-01-21

**Authors:** Lucía Pedrosa, Ismael Fernández-Miranda, David Pérez-Callejo, Cristina Quero, Marta Rodríguez, Paloma Martín-Acosta, Sagrario Gómez, Julia González-Rincón, Adrián Santos, Carlos Tarin, Juan F. García, Francisco R. García-Arroyo, Antonio Rueda, Francisca I. Camacho, Mónica García-Cosío, Ana Heredero, Marta Llanos, Manuela Mollejo, Miguel Piris-Villaespesa, José Gómez-Codina, Natalia Yanguas-Casás, Antonio Sánchez, Miguel A. Piris, Mariano Provencio, Margarita Sánchez-Beato

**Affiliations:** 1 Lymphoma Research Group, Medical Oncology Department, Instituto de Investigación Sanitaria Puerta de Hierro-Segovia de Arana, Majadahonda, Madrid Spain; 2 PhD Program in Molecular Biosciences, Doctoral School, Universidad Autónoma de Madrid, Madrid, Spain; 3 Medical Oncology Department, Hospital Universitario Puerta de Hierro-Majadahonda, Madrid, Spain; 4 PhD Program in Medicine and Surgery, Doctoral School, Universidad Autónoma de Madrid, Madrid, Spain; 5 Medical Oncology Department, Hospital Universitario Virgen de La Victoria, Malaga, Spain; 6 Pathology Department, Hospital Fundación Jiménez Díaz, Madrid, Spain; 7 Centro de Investigación Biomédica en Red de Cáncer (CIBERONC), Madrid, Spain; 8 Molecular Pathology Laboratory, Instituto de Investigación Sanitaria Puerta de Hierro-Segovia de Arana, Madrid, Spain; 9 Bioinformatics Unit, Instituto de Investigación Sanitaria Puerta de Hierro-Segovia de Arana, Madrid, Spain; 10 Basic Medical Sciences, Faculty of Medicine, Universidad CEU San Pablo, Madrid, Spain; 11 Pathology Department, MD Anderson Cancer Center, Madrid, Spain; 12 Medical Oncology Department, Complejo Hospitalario de Pontevedra, Pontevedra, Spain; 13 Medical Oncology Department, Hospitales Universitarios Regional y Virgen de La Victoria, IBIMA, Malaga, Spain; 14 Pathology Department, Hospital Universitario de Getafe, Madrid, Spain; 15 Pathology Department, Hospital Universitario Ramón y Cajal, Madrid, Spain; 16 Medical Oncology Department, Hospital Universitario de Canarias, Tenerife, Spain; 17 Pathology Department, Complejo Hospitalario de Toledo, Toledo, Spain; 18 Haematology Department, Hospital Universitario Ramón y Cajal, Madrid, Spain; 19 Medical Oncology Department, Hospital Universitari i Politècnic La Fe, Valencia, Spain

**Keywords:** Lymphoma, Molecular medicine, Next-generation sequencing

## Abstract

Diffuse large B-cell lymphoma (DLBCL) is a heterogeneous disease whose prognosis is associated with clinical features, cell-of-origin and genetic aberrations. Recent integrative, multi-omic analyses had led to identifying overlapping genetic DLBCL subtypes. We used targeted massive sequencing to analyze 84 diagnostic samples from a multicenter cohort of patients with DLBCL treated with rituximab-containing therapies and a median follow-up of 6 years. The most frequently mutated genes were *IGLL5* (43%), *KMT2D* (33.3%), *CREBBP* (28.6%), *PIM1* (26.2%), and *CARD11* (22.6%). Mutations in *CD79B* were associated with a higher risk of relapse after treatment, whereas patients with mutations in *CD79B*, *ETS1,* and *CD58* had a significantly shorter survival. Based on the new genetic DLBCL classifications, we tested and validated a simplified method to classify samples in five genetic subtypes analyzing the mutational status of 26 genes and *BCL2* and *BCL6* translocations. We propose a two-step genetic DLBCL classifier (2-S), integrating the most significant features from previous algorithms, to classify the samples as N1^2-S^, EZB^2-S^, MCD^2-S^, BN2^2-S^, and ST2^2-S^ groups. We determined its sensitivity and specificity, compared with the other established algorithms, and evaluated its clinical impact. The results showed that ST2^2-S^ is the group with the best clinical outcome and N1^2-S^, the more aggressive one. EZB^2-S^ identified a subgroup with a worse prognosis among GCB-DLBLC cases.

## Introduction

Diffuse large B-cell lymphoma (DLBCL) is the most common subtype of non-Hodgkin lymphoma, with 3.13 and 5.6 new cases diagnosed in Europe^1^ and the USA^2^ per 100,000 habitants per year, respectively. DLBCL is an aggressive and heterogeneous disease with a variable clinical outcome; it can arise de novo or after histological transformation from other low-grade lymphomas, typically from follicular lymphoma. Most DLBCL patients can be cured by standard immunochemotherapy with R-CHOP (rituximab, cyclophosphamide, doxorubicin, vincristine, and prednisone)^3^. However, a substantial percentage of them (30–40%) are refractory to treatment or relapse (R/R) after an initial response, and it is not possible to accurately predict which patients will benefit from rituximab-based therapy^4^. Currently, the International Prognostic Index (IPI), based on clinical and analytical characteristics, is the most significant predictive factor, in which higher scores are associated with unfavorable outcomes^5–7^.

The most recent revision of the WHO classification of lymphoid neoplasms recognizes the high-grade B-cell double-/triple-hit lymphoma (DH/TH), with *MYC* and *BCL2* and/or *BCL6* rearrangements, as a new provisional entity associated with an inferior outcome. DLBCLs co-expressing MYC and BCL2 (double-expressor lymphomas) also have a worse prognosis than other DLBCL-NOS (not otherwise specified), but their behavior is not as aggressive as that of DH/TH lymphomas^8^.

Gene-expression profiling (GEP) allows distinguishes three subtypes based on cell of origin (COO): germinal center B-cell (GCB)-like, activated B-cell (ABC)-like and unclassified subtypes^9,10^. This classification has been shown to be of prognostic value, with ABC-DLBCL being associated with poorer outcome^9–11^, but it does not fully explain the high DLBCL heterogeneity, or accurately predict the response to standard therapy. The truth is that all patients are treated identically, independently of their COO subtype. Therefore, we need to identify the genetic alterations of DLBCL associated with refractoriness and develop alternative treatments or novel pharmacological strategies to overcome this resistance.

In the last few years, deep-sequencing studies have allowed a better understanding of the DLBCL genomic landscape and provided further evidence of their molecular heterogeneity. Several recent studies have proposed new genetic subtypes based on the DLBCL genomic profile. Although they are somewhat different, the newly defined genetic subtypes share several characteristics. Schmitz and colleagues identified four genetic subgroups, which they referred to as MCD (characterized by the co-occurrence of *MYD88*^L265P^ and *CD79B* mutations), BN2 (with *BCL6* fusions and *NOTCH2* mutations), N1 (with *NOTCH1* mutations) and EZB (characterized by *EZH2* mutations and *BCL2* translocations). MCD and N1 are associated with poorer outcomes than the other subtypes^12^. Most recently, the same group developed the LymphGen algorithm, which allows a more precise genetic classification, adding the A53 (characterized by *TP53* mutations and deletions) and ST2 (*SGK1* and *TET2* mutated) subtypes, to the previous ones^13^. Chapuy et al.^14^ distinguished five subsets of DLBCL, including two ABC-DLBCL groups, one with low risk and a possible marginal zone origin (C1), and the other a high-risk group (C5) enriched in cases with mutations in *MYD88*, *CD79B*, and *PIM1*; they also described two subsets of GCB-DLBCLs with favorable (C4) and poor (C3) outcomes, and an ABC/GCB-independent group (C2) with biallelic inactivation of *TP53*, *CDKN2A* loss, and associated genomic instability. Finally, in an attempt to bring these genetic classifications together, Lacy et al*.*^15^ characterized five molecular subtypes, NOTCH2, MYD88, BCL2, TET2/SGK1, and SOCS1/SGK1, according to the mutated genes that are most highly enriched in each. The genetic classifications partially overlap, suggesting the existence of accurate molecular subtypes that might have predictive and prognostic capability and help to select the most appropriate therapy for each DLBCL patient. However, for these genetic classifications to be genuinely useful, a feasible, consensus classification based on selected genetic alterations must be validated to contribute to a sensitive and specific classification that is of clinical value.

We propose a simplified classification based on the previous ones, validated using data obtained by targeted deep-sequencing analysis in a set of diagnostic samples from DLBCL patients treated with R-CHOP or similar regimens, 40% of whom (34/84) did not respond, or relapsed after treatment.

## Results

### Mutational profile and association with clinical outcome

Patient and tumor characteristics of the "Puerta de Hierro" (PdH) cohort (n = 84) are shown in Table 1 and Supplementary Table S1. FISH analysis and COO classification were performed on samples with available material. Two of the 44 samples studied by FISH (4.5%) were classified as DH. Regarding COO, Lymph2Cx-based assay determined that 53.7% (29/54) of the cases were GCB, 25.9% ABC (14/54) and 20.4% (11/54) unclassified, while the Hans algorithm classified 50.8% (33/65) as GCB and 49.2% (32/65) as non-GCB.

**Table 1:** Summary of clinical data of the Puerta de Hierro (PdH) cohort.

Clinical variable	N	Categories	N	Percentage
Sex	84	Male	46	54.8
		Female	38	45.2
Age	83	≥ 60	47	56.7
		< 60	36	43.4
ECOG	78	0	28	36.8
		1	29	38.2
		2	18	23.7
		3	3	3.9
		4	0	0
		5	0	0
Stage	81	I	6	7.8
		II	23	29.9
		III	31	40.3
		IV	31	40.3
IPI	83	Low	28	33.7
		Low-Intermediate	23	27.7
		High-Intermediate	18	21.7
		High	14	16.9
Treatment	84	R-CHOP	76	90.5
		R-like	8	9.5
Response to treatment	83	Complete response	66	79.5
		Partial response	6	7.2
		No response	11	13.3
Refractory/Relapse	84	Refractory/Relapse	34	40.5
		No relapse	50	59.5
Status	84	Exitus	33	39.3
		Alive	51	60.7
COO (Lymph2Cx)	54	GCB	29	53.7
		ABC	14	25.9
		Unclassified	11	20.4
COO (Hans)	65	GCB	33	50.8
		Non-GCB	32	49.2
FISH		BLC2	11/50	22.0
		BCL6	18/45	40.0
		MYC	5/49	10.2
		Double-HIT	2/44	4.5

International Prognostic Index (IPI), rituximab, cyclophosphamide, doxorubicin, vincristine, and prednisone (R-CHOP), rituximab-like (R-like), cell of origin (COO), activated B-cell (ABC), germinal center B-cell (GCB), fluorescence in situ hybridization (FISH).

**Figure 1:**
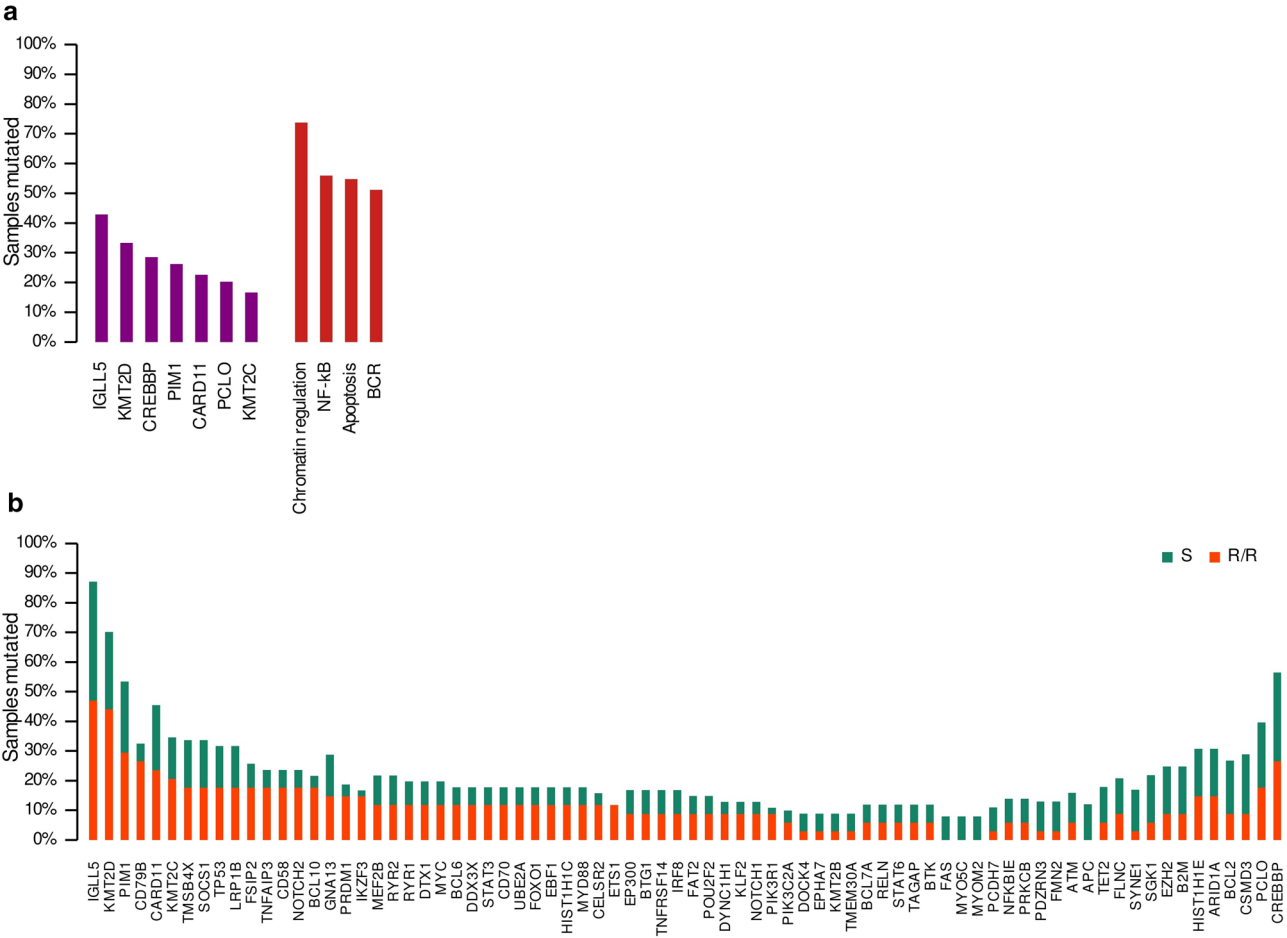
Mutational prevalence in genes and pathways. (**A**) Most frequently mutated genes and pathways in the whole cohort. (**B**) Mutational prevalence for refractory/relapsed (R/R) and sensitive (S) cases for genes with more than four samples mutated.

We performed targeted massive parallel sequencing in the 84 DLBCL samples, and were able to identify at least one somatic mutation in all the samples (Suppl. Table S2). All the genes included in the panel (125; see “Methods” section) were mutated at least once, except *KRAS* and *CD22*. A total of 1030 somatic mutations (SNVs and indels) were detected, considering missense, non-sense, and splicing mutations. The samples harbored a median of 12.3 mutations (range: 2–45), 13.4 mutations in R/R cases (range: 2–45), and 11.5 mutations in sensitive (S) cases (range: 3–30). The most recurrently mutated genes were *IGLL5* (42.9%, 36/84 cases), *KMT2D* (33.3%, 28/84), *CREBBP* (28.6%, 24/84), *PIM1* (26.2%, 22/84), *CARD11* (22.6%, 19/84), *PCLO* (20.2%, 17/84), and *KMT2C* (19.4%, 13/84) (Fig. 1A; Suppl. Table S3).

We also explored the DLBCL mutational landscape in predefined signaling pathways or lymphomagenesis-related gene sets. Genes included in every gene set, based on the previously published B-cell NHL gene signatures^16^, are summarized in Supplementary Table S4. Overall, the samples had a higher incidence of mutations in genes involved in chromatin regulation (73.8%, 62/84), NFκB (58.3%, 47/84), apoptosis (54.8%, 46/84), and BCR pathways (51.2%, 43/84) (Fig. 1A).

We assessed the prognostic value of IPI and the COO classification in our series. The IPI predicted shorter overall survival (OS) (Intermediate risk: p = 0.009, HR = 5.04; High risk: p = 0.003; HR = 7.9) and progression-free survival (PFS) (Intermediate risk: p = 0.002, HR = 6.72; High risk: p = 0.004; HR = 7.13) by Cox proportional-hazards model analysis for the intermediate- and high-risk groups. When we evaluated the prognostic value of the COO by the Kaplan–Meier survival method, GCB cases showed better clinical outcomes, but the magnitude of the differences was not great enough to be significant, given the small number of classified samples available (Fig. S1).

**Figure 2:**
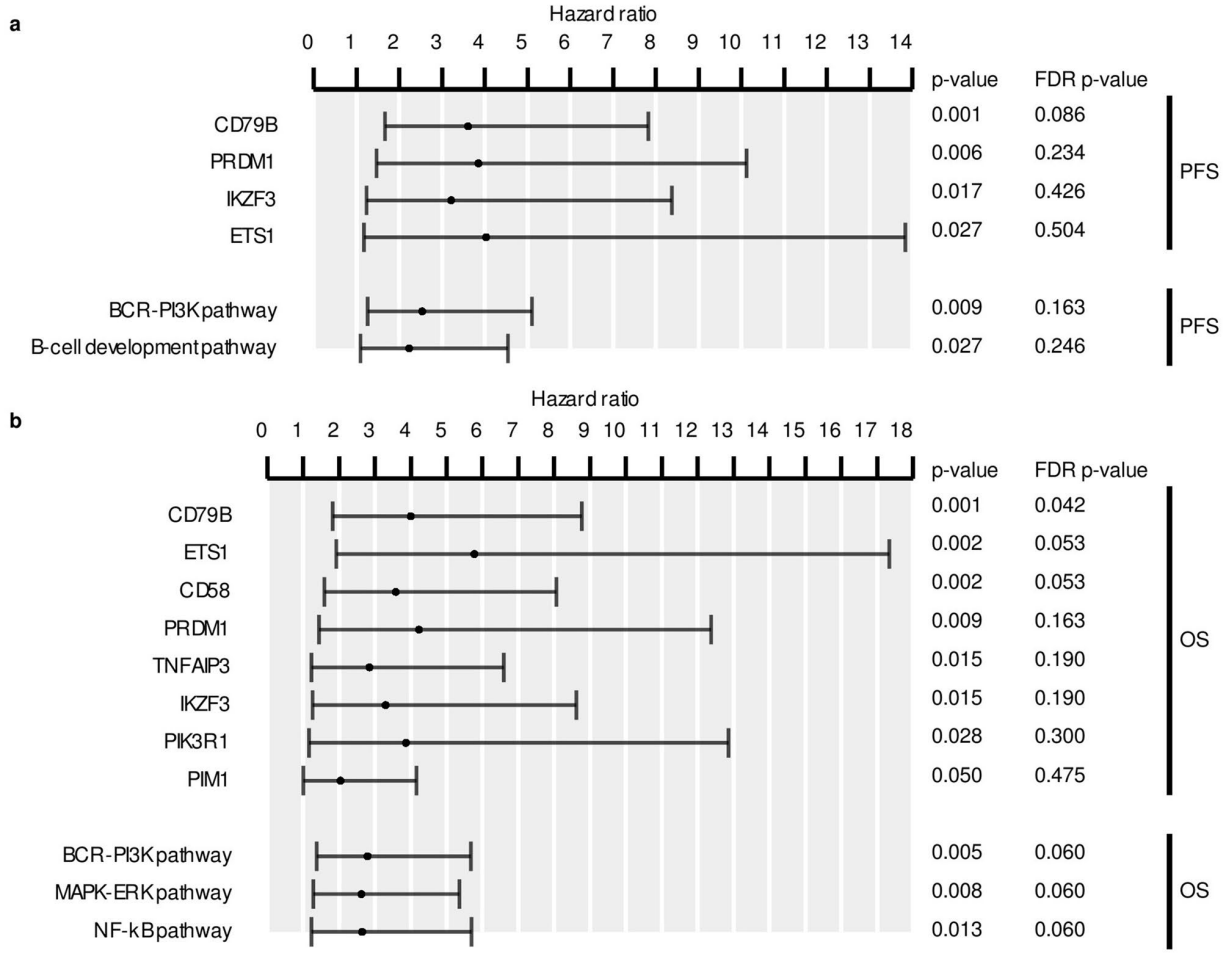
Statistical analysis of genes and pathways in the PdH cohort. Mutated genes and pathways with significant p-values in the univariate Cox proportional-hazards model analysis for (**A**) progression-free (PFS) and (**B**) overall survival (OS). Error bars represent the 95% confidence intervals for the hazard ratios. FDR p-values for Benjamini–Hochberg correction are shown.

We then explored the clinical relevance of the gene/pathway mutations present in at least four samples. The analysis showed that *CD79B* mutations were associated with a higher risk of R/R (Figs. 1B, 2A). Similarly, patients with mutations in B-cell development and BCR-PI3K pathways were more prone to relapse after therapy. Although not significantly associated, several other genes and pathways were more frequently mutated in samples from R/R patients, such as *PRDM1* (15% *vs*. 4%), *ETS1* (12% *vs*. 0%), *IKZF3* (15% *vs*. 2%), *BCL10* (18% *vs.* 4%), *NOTCH2* (18% *vs*. 6%), *CD58* (16% *vs. 6*%), and MAPK-ERK (32% *vs.* 16%), and Toll pathways (18% *vs.* 8%) (Fig. 1B, Suppl. Tables S3 and S5). Furthermore, the multivariate analysis, combined with the IPI, showed that patients with mutations in *CD79B* had an independent association with shorter PFS (HR = 2.73, p = 0.01). The analysis also demonstrated that mutations in *CD79B*, *ETS1*, and *CD58*, were associated with shorter OS (Fig. 2B), as well as mutations in the BCR-PI3K, MAPK-ERK, and NFκB pathways (Fig. 2B). Mutations in *CD79B* (HR = 2.72, p = 0.02), and *ETS1* (HR = 3.67, p = 0.03) were also associated with a worse OS, as indicated by the multivariate analysis with the IPI.

### Proposal of a combined two-step method for DLBLC genetic classification

Several attempts have been made to improve COO classification by considering tumor genetics in order to facilitate the use of precision-medicine approaches in DLBLC patients. This has led to new DLBCL genetic classifications, as described earlier. One of our aims was to look for a method to unify them and simplify the classification algorithms in such a way as to facilitate its routine use in pathology laboratories eventually to enable prospective sample classification. For this purpose, we developed a classification method based mainly on the Wright^13^ and Lacy^15^ subtypes characteristics, integrating the most significant features from both studies. To refine and evaluate our method, we used the United Kingdom population-based Haematological Malignancy Research Network (HMRN) cohort^15^, restricting our analysis to DLBLC-NOS patients treated with R-CHOP (n = 580).

**Figure 3:**
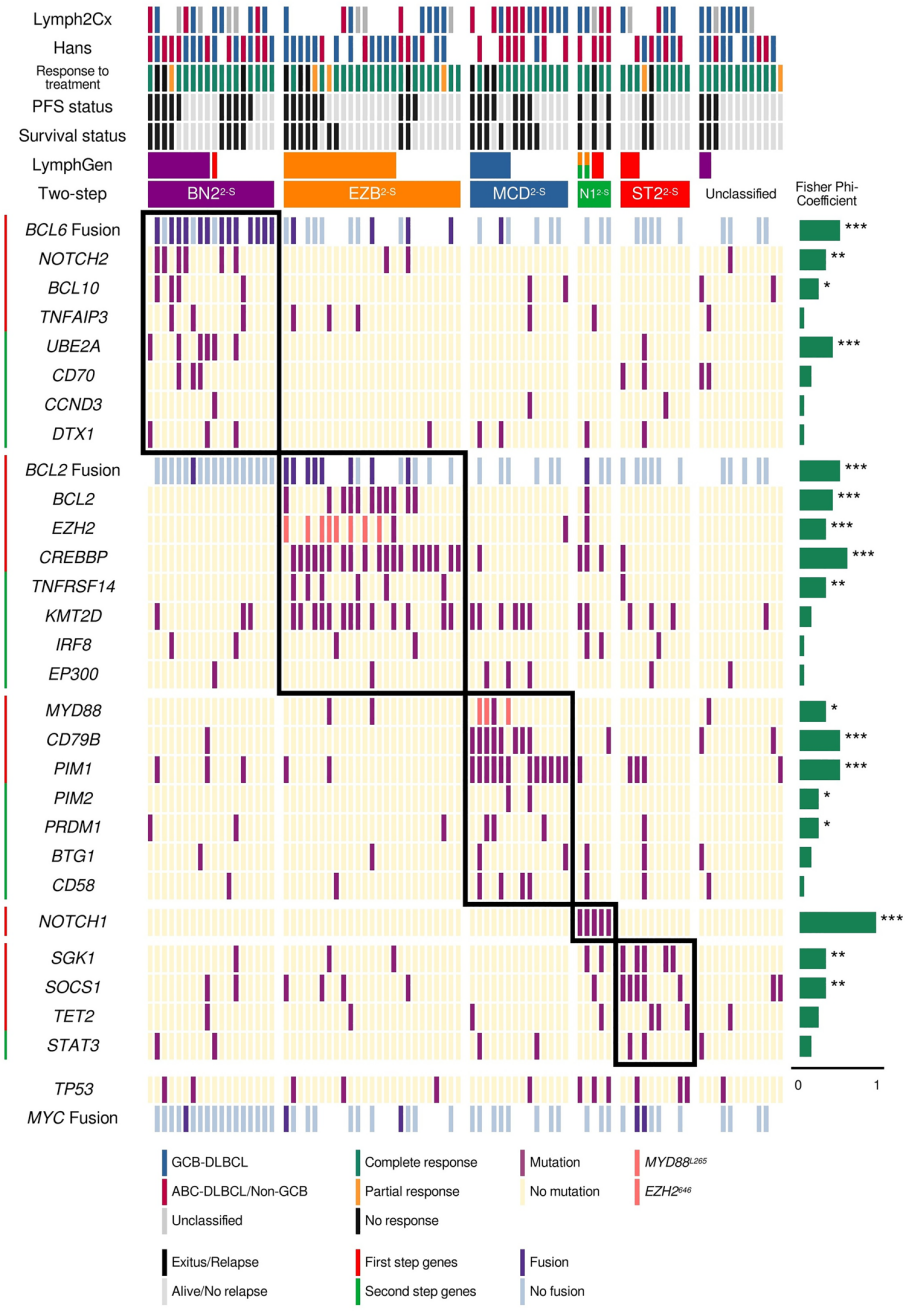
Genetic subtypes and association with overall survival (OS) and progression-free survival (PFS). Genetic classification in the five defined genetic subtypes of DLBCL. Clustering was performed using alterations in genes (rows) from 84 DLBCL samples (columns). The OS and event-free survival status, and the ABC/GCB classification based on Lymph2cx and Hans are represented at the top. The phi coefficient and Fisher's exact test significance are represented on the right of the figure (*p < 0.05; **p < 0.01; ***p < 0.001).

Several tests were carried out to select the best combination of genes, taking into account the sensitivity and specificity compared with the PdH and HMRN cohorts using the LymphGen algorithm^13^ (see “Methods” section). We selected the combination of genes to be used in the two-step (2-S) classifier, and then applied the approach to our cohort. Five cases were classified as N1^2-S^, 18 as BN2^2-S^, 25 as EZB^2-S^, 14 as MCD^2-S^, and ten as ST2^2-S^. Twelve cases (14.3%) remained unclassified (Fig. 3).

When we classified the PdH cohort using LymphGen, 11 cases were assigned as BN2, 16 as EZB, six as MCD, and six as ST2. Although we have five cases with *NOTCH1* mutations, only two were assigned as EZB/N1 complex genotype by LymphGen. Forty-three cases (53%) remained unclassified. Compared with LymphGen, the two-step classifier showed a 100% sensitivity for the MCD^2-S^ and EZB^2-S^, 81.8% for the BN2^2-S^, and 50% for the ST2^2-S^ subtypes, taking the LymphGen classification as reference (Table 2; Fig. 3). The specificity values achieved were 89.7% for MCD^2-S^, 88% for BN2^2-S^, 86.8% for EZB^2-S^, and 91.4% for ST2^2-S^ (Table 2; Fig. 3).

**Table 2:** Sensitivity and specificity analysis comparing the three classifiers in the Puerta de Hierro (PdH) and HMRN cohorts.

	Sensitivity/specificity (%)									
	MCD^2-S^		BN2^2-S^		EZB^2-S^		ST2^2-S^		N1^2-S^	
**PdH cohort**										
Two-step versus LymphGen	100	89.7	81.8	88.0	100	86.8	50	91.4	–	94.1
Two-step versus AIC cluster	97.5	92.0	41.2	97.2	88.5	90.6	56.1	95.8	–	–
**HMRN cohort**										
Two-step versus LymphGen	98.5	90.1	64.0	95.8	89.9	92.8	81.3	92.7	100	99.8
LymphGen versus AIC cluster	81.3	99.6	39.2	97.8	84.9	93.5	50.4	99.0	–	–

**Figure 4:**
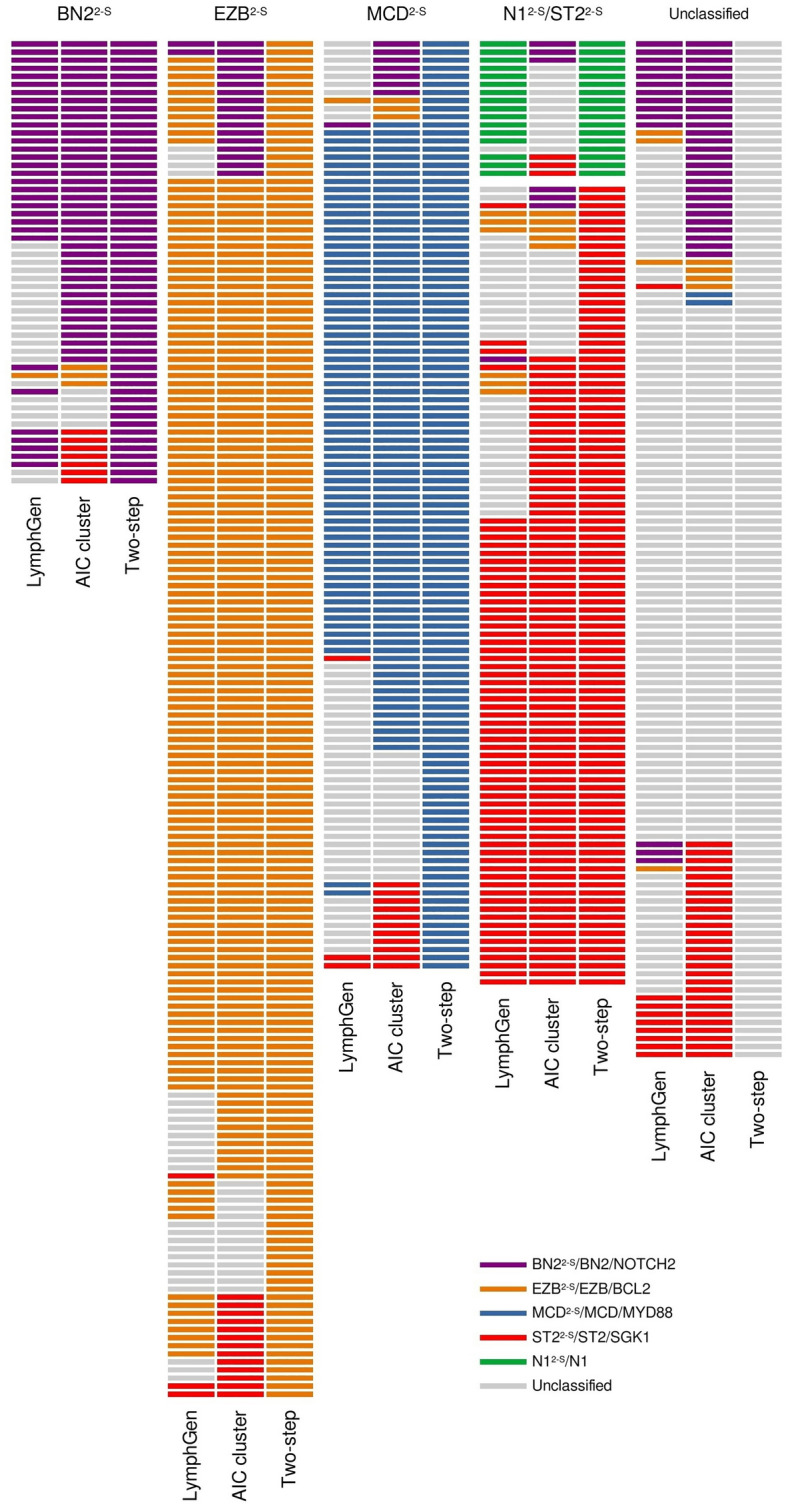
Comparison of the classifiers. Subtypes assigned to the HMRN cohort by LymphGen, AIC cluster and the two-step classifier.

Due to the small size of our cohort, to validate the method, we applied the two-step as well as the LymphGen classifiers to the HMRN cohort, and compared them to the Akaike Information Criterion (AIC) clustering method employed in the original study^15^. The sensitivity values for the two-step classifier compared with the AIC cluster were 97.5% for MCD^2-S^, 41.2% for BN2^2-S^, 88.5% for EZB^2-S^, and 56.1% for ST2^2-S^. Regarding specificity, all the subtypes achieved values greater than 90% (Table 2; Fig. 4). No sensitivity and specificity values were obtained for N1^2-S^ because the AIC cluster did not include this group. The values comparing the two-step classification to LymphGen were similar, except for the BN2^2-S^/BN2 and ST2^2-S^/ST2, with 64.0 and 81.3% sensitivity, respectively (Table 2; Fig. 4). Seventeen samples were classified as N1^2-S^ by our classifier, 16 of them also ranked as N1 by LymphGen. BN2/NOTCH2 and ST2/SGK1-TET2-SOCS1 also showed differences between with sensitivity values of 39.2 and 50.4%, respectively (Table 2; Fig. 4).

The low sensitivity of the BN2^2-S^ subtype prompted us to analyze the samples included in this cluster more thoroughly. Twenty-eight percent of NOTCH2 cases in the HMRN cohort remained unclassified by our two-step classifier. Eighteen percent had more mutations in genes for the EZB^2-S^ subtype and were, therefore, classified as such. Similar low sensitivity was obtained when compared with the LymphGen classification, in which 28% of the BN2 cases in the HMRN cohort were not assigned to any subtype by the two-step method. When we compared the AIC cluster with the LymphGen, 43% of the NOTCH2 cases were unclassified by LymphGen, and 13% were assigned to EZB.

When we examined the new two-step classifier's potential clinical value, the survival analyses produced no significant results in the PhD cohort, although they did reveal a trend towards shorter OS and higher risk of relapse for MCD^2-S^ and BN2^2-S^ cases compared with EZB^2-S^ and ST2^2-S^ (Fig. S2A). MCD was the subtype with the worst OS and PFS prognosis for the LymphGen classification, although, again, the differences were not substantial enough to be statistically significant, given the small number of classified samples by both methods (67 and 39, respectively) (Fig. S2B).

**Figure 5:**
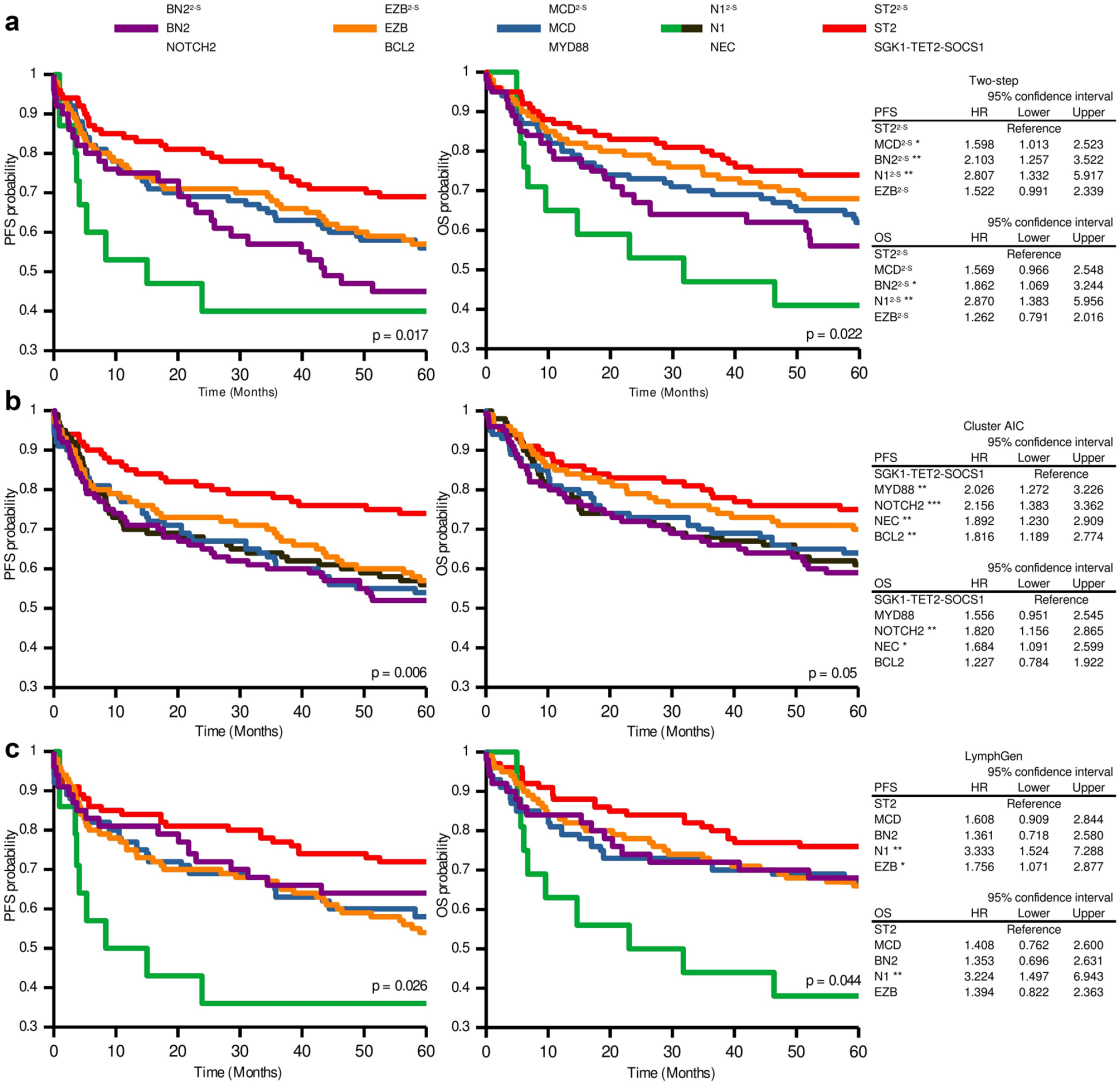
Progression-free survival (PFS) and overall survival (OS) according to the genetic subtypes of the two-step, AIC cluster and LymphGen classifiers in the HMRN cohort. Kaplan–Meier analysis of genetic subtypes from (**A**) two-step, (**B**) AIC cluster and (**C**) LymphGen methods. Tables on the right of the figure show the hazard ratio (HR) values from the Cox proportional-hazards model of genetic subtypes for PFS and OS status. Error bars represent the 95% confidence intervals. Significance: *p < 0.05; **p < 0.01.

Therefore, we evaluated the two-step classifier's clinical relevance using data from the DLBLC-NOS patients treated with R-CHOP included in the HMRN cohort. The analysis assigned a higher risk of relapse to the N1^2-S^, BN2^2-S^, and MCD^2-S^ cases than to the ST2^2-S^ cases (Fig. 5A). N1^2-S^ and BN2^2-S^ also had shorter OS. We compared these results with those obtained by the original AIC cluster and LymphGen classifiers, and we found that the groups defined by the three methods had *grosso modo* similar associations with PFS and OS, as indicated by Kaplan–Meier and Cox proportional-hazards analysis (Fig. 5A–C). ST2^2-S^/ST2/SGK1-TET2-SOCS1 is the group with the best clinical outcome. N1^2-S^ and N1 showed the shortest OS (Fig. 5A,C).

The three classifiers showed similar PFS and OS for MCD^2-S^/MCD/MYD88, EZB^2-S^/EZB/BCL2, and ST2^2-S^/ST2/SGK1-TET2-SOCS1 subtypes, as demonstrated by the superimposed curves from the Kaplan–Meier analysis (Fig. S3). BN2^2-S^/BN2/NOTCH2 is the group that differed the most depending on the classifier used, showing shorter OS and PFS with the two-step classification and the AIC cluster compared with LymphGen (Fig. S3A).

**Figure 6:**
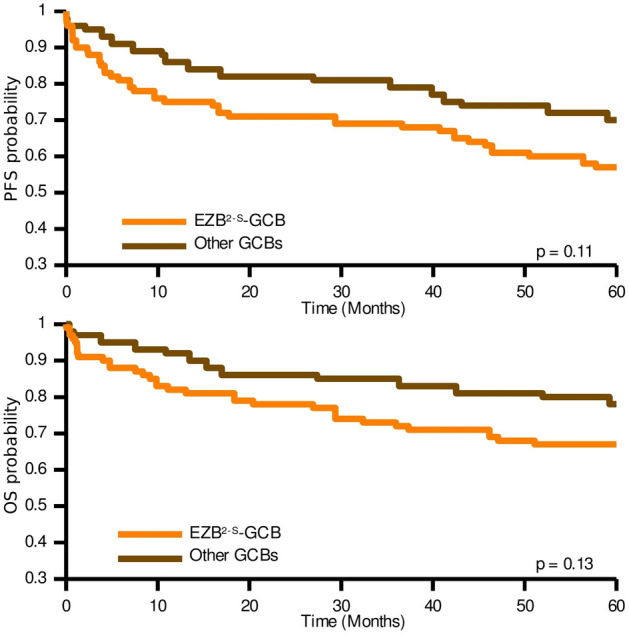
Kaplan–Meier analysis of progression-free survival (PFS) and overall survival (OS) for EZB^2-S^-GCB and other GCB cases in the HMRN cohort when applying the two-step classifier.

Among the GCB-DLBLC cases, EZB^2-S^ cases showed worse clinical outcomes, although not statistically significant (Fig. 6). The 36% of the non-EZB^2-S^ GCB-DLBCL cases were classified as ST2^2-S^, 12% as MCD^2-S^, 11% as BN2^2-S^; 40% of samples were unclassified. No differences were found between subtypes in the ABC-DLBLC cases.

Finally, the predictive value of the subtypes in combination with the IPI was analyzed. We performed a multivariate Cox proportional-hazards analysis with the IPI and the two-step classifier-defined genetic subtypes in the HMRN cohort (Table S6), and subsequently validated the results in the PdH cohort. This model assigned progression and survival risk scores for each sample in the PdH cohort (Table S1), from which we generated three risk groups with different clinical outcomes, as indicated by the Kaplan–Meier analysis (p = 0.038 for OS; p = 0.005 for PFS) (Fig. S4).

## Discussion

Our study confirmed the current landscape of genetic alterations in DLBCL and explored their involvement in clinical outcome and response to treatment. We also propose a modified genetic classification based on those of the Wright et al.^13^ and Lacy et al.^15^, validated its accuracy, and analyzed its ability to predict response to therapy and clinical outcome in R-CHOP-treated DLBCL patients.

Mutational data confirmed the well-known high degree of heterogeneity in DLBCLs. We found that the genes most frequently mutated in this cohort were *IGLL5*, *KMT2D*, *CREBBP*, *PIM1*, *CARD11*, *PCLO*, and *KMT2C*, similar to what has been noted in other DLBCL series^12,14,17–21^. When we integrated the genes in predefined signaling pathways or gene functions, chromatin remodeling, BCR, NFκB, and apoptosis pathways were the most frequently mutated gene sets (Fig. 1A).

The main problems for DLBCL patients are the high percentage of them (30–40%) that are refractory to treatment or relapse, and the lack of accurate predictive markers. We found that mutations in *CD79B* were associated with relapse, independent of the IPI. We also tested whether any alteration was related to primary refractoriness, but none was found to be enriched in samples from R/R patients (data not shown). Patients with mutations in *CD79B, ETS1,* and *CD58* had significantly lower survival rates. *CD79B* and *CD58* mutations had previously shown adverse prognostic value, and *CD79B* was already known to be one of the most frequently mutated genes at relapse^22–25^. Mutations in *ETS1* were found more frequently in Burkitt lymphoma, but only sporadically in DLBLC. ETS1 regulates B-cell-relevant pathways, such as B-cell differentiation. ETS1 overexpression has been associated with gain of the 11q24.3 region. This region, characteristic of a Burkitt-like lymphoma subtype (BLL-11q), has also been found to be gained in up to a quarter of DLBCL cases^26,27^.

Several recent studies have undertaken integrative, multiplatform analysis of gene mutations, structural aberrations, and gene-expression profiling^12,13,15,28,29^ that have led to the proposal of new genetic subtypes determined by distinct genetic backgrounds and clinical behaviors. Although these classifications share several characteristics and genetic features, their different uses lead to some inconsistencies in determining definite clusters. For example, Schmitz^12^ and Wright^13^ and their colleagues used *BCL2* and *BCL6* fusions, essential drivers of DLBCL pathogenesis, as classifier alterations, whereas Lacy et al.^15^ did not use them to classify their samples; on the other hand, Chapuy et al.^14^ used CNVs to define their clusters, which are especially relevant for the C2 subtype. The algorithms' complexity and the large number of genes and structural alterations used to define the genetic subtypes made it challenging to use them as part of the clinical routine. Moreover, some classifications, like LymphGen^13^, produce a high percentage of unclassified tumors. Others, such as that of Chapuy^14^ and Lacy^15^ and their coworkers, could not be used for individual samples. Here, we propose an easier classifier, using a panel with a small number of genes (26) and *BCL2* and *BCL6* translocations detected by routine FISH technique, to define the genetic subtypes. This two-step classifier is based mainly on the approaches of Wright^13^ and Lacy^15^, and uses the most frequently mutated genes shared by the two studies, maintaining the names given by Schmitz^12^ and Wright^13^. We define five groups and classify the samples as N1^2-S^, MCD^2-S^, BN2^2-S^, EZB^2-S^, or ST2^2-S^.

Given the limitations of this analysis arising from the low number of patients, we applied it in the external HMRN series, restricting the analyses to DLBCL-NOS patients treated with R-CHOP.

Despite the limitations of the study, the comparison of the two-step classifier specificity and sensitivity with that of LymphGen and the AIC cluster, and assessing its prognostic and predictive value in a large real-world series (HMRN), showed our method to be robust and demonstrated that the clinical outcomes of the subgroups did indeed differ.

N1^2-S^ was defined by the presence of *NOTCH1* mutations regardless of all other alterations detected in the tumors. Although N1 was not included in the Lacy classification, we considered it an essential subtype because it is the most aggressive (Fig. 5), as previously found by Schmitz^12^ and Wright^13^, and demonstrated in the Lacy series^15^.

The BN2^2-S^ group had the worst clinical outcome of our series, with results similar to those of Lacy^15^, but unlike those of Chapuy^14^ and of Schmitz–Wright^12,13^ (Fig. S2). The associations of the BN2 and C1 subtypes with a more favorable outcome were explained as being a possible transformation from occult MZL. The BN2-NOTCH2-C1 subtype was more weakly defined than the others, probably due to the disagreement regarding the use of *BCL6* translocations and the selection of genes defining it. They are based on several unequally shared characteristics, such as *NOTCH2* (shared by Schmitz–Wright^12,13^ and Lacy^15^), *BCL10* (Schmitz–Wright^12,13^ and Chapuy^14^) or *BCL6* translocations (Lacy^15^ and Chapuy^14^). These differences may explain the smaller overlap and the different clinical behavior in the three series. We decided to include *BCL2* and *BCL6* translocations due to their importance in DLBCL pathogenesis, the availability of the data for most DLBCL samples, the strong relationship between *BCL2* mutations and BCL2 translocation, and the fact that, in the Lacy series^15^, the NOTCH2 subtype was enriched in samples with *BCL6* fusions.

ST2^2-S^/ST2/SGK1-TET2-SOCS1 was the subtype with the best clinical outcome, as previously reported^13,15^, and confirmed here with the three classifiers. MCD^2-S^/MCD/ MYD88 and EZB^2-S^/EZB/BCL2 are the most consistent subtypes, as shown by the high sensitivity and specificity values when comparing the three classifiers and the overlap of the Kaplan–Meier curves (Fig. 5 and Fig. S3). Comparing EZB^2-S^ with other GCB cases (Fig. 6) revealed the former to have a slightly worse clinical outcome.

Finally, given the limitations of analyzing CNVs in standard pathology laboratories, we decided not to include A53 (Wright^13^)/C2 (Chapuy^14^) in our classifier as they were identified by *TP53* mutations and deletions together with widespread CNVs.

The original classifiers are of limited use in clinical practice because they are based on so many genetic alterations and such complex algorithms. Here we propose a genetic DLBCL classifier that integrates the findings of Schmitz–Wright^12,13^ and Lacy^15^ and that is based on an optimized panel with a minimal set of markers (26 genes and the *BCL2* and *BCL6* translocations) that can classify samples individually for the purpose of routine patient management. The validations carried out in this study demonstrated the close correlation between the two-step classifier and the other classifiers, as the specificity and sensitivity analysis showed it to be adequate for achieving a successful classification.

Despite the well-known heterogeneous clinical behavior of DLBCL patients, the diagnostic work-up and treatment are identical for every patient. Although COO classification provided significant prognostic information, and some targeted therapies have shown different efficacy depending on the COO, this has not led to any changes in the use of R-CHOP as the standard treatment for DLBLC patients. We hope that this classifier would make it possible to assign patients to a genetic subtype on a tumor-by-tumor basis, thereby guiding the clinical management of individual patients.

The genetic alterations defining each subtype, the association of functionally altered pathways, and previously performed functional studies suggested the appropriate therapeutic approach for each of them. N1 is the most interesting due to its poor response to standard immunochemotherapy. *NOTCH1* aberrations frequently occur in hematological malignancies, mainly chronic lymphocytic leukemia and T-cell leukemia, making this gene a candidate target in the design of tailored therapy for this subtype. In this context, γ-secretase inhibitors (GSIs) are the most widely studied anti-NOTCH1 molecules in cancer. The clinically relevant GSI PF-03084014, combined with fludarabine, has antitumor effects in primary *NOTCH1*-mutated CLL cells^30^ and, when tested in phase I clinical trials, showed good tolerance and antitumor activity in solid and hematological malignancies^31,32^. Therefore, the combination of GSIs with chemotherapy is a promising strategy for treating N1 DLBCL patients. Several approaches have already been suggested for the other subtypes, such as BTK inhibitors for MCD cases, EZH inhibitors, and BCL2 inhibitors, such as venetoclax or navitoclax, for the EZB subtype^13^.

In conclusion, we propose a feasible classifier built with mutation information from 26 genes and *BCL2/BCL6* translocations, based on the recently identified genetic subgroups^12–15^. This genetic classifier, combined with clinical data and other molecular characteristics, should eventually help researchers develop improved risk models for DLBCL patients and, more importantly, for guiding precision therapy.

## Methods

### Patients and samples

The study population (PdH cohort) consisted of 84 DLBCL patients, diagnosed in Spanish medical institutions between 2002 and 2016. Five of the patients (PDLB63-PDLB67) had a diagnosis of DLBCL after a histological transformation of follicular lymphoma without treatment prior to transformation^33^. Formalin-fixed, paraffin-embedded tissue (FFPET) sections from diagnostic biopsies were collected. The research project was approved by the Ethics Committee of Hospital Universitario Puerta de Hierro-Majadahonda (reference PI67-14) and conducted in compliance with the Declaration of Helsinki. All participants signed an informed consent form. Samples were collected and clinical data managed following protocols guaranteeing the confidentiality of donor data. Material and data from other centers were anonymously transferred to our laboratory after obtaining approval from the corresponding ethics committees, under the relevant Spanish legislation (*Ley 14/2007 de Investigación Biomédica* and *Real Decreto 1716/2011*). All samples were reviewed upon arrival by qualified hematopathologists to confirm their diagnosis.

Patients were treated with R-CHOP (77 patients) or R-CHOP-like (7 patients) regimens. The retrospective collection included FFPET sections from samples at diagnosis from 50 patients (59.5%) who responded to treatment, referred to hereafter as sensitive (S), and 34 samples (40.5%) from refractory (11 patients) and relapsed (23) (R/R) patients. The median follow-up was 6 years. For the IPI analysis, we reclassified the patients into three risk categories: low (0–1), intermediate (2–3) and high (4–5). Patient characteristics are summarized in Table 1 and Suppl. Table S1.

### Targeted sequencing

Genomic DNA was extracted from FFPET using a truXTRAC FFPE DNA Kit (Covaris, Woburn, MA, USA) following the manufacturer's instructions.

Two SureSelect target enrichment custom panels were designed using the SureDesign (Agilent Technologies, Santa Clara, CA, USA) web-based tool (earray.chem.agilent.com/suredesign/). The genes included are involved in lymphomagenesis-relevant pathways and were selected based on previous studies^17,18,34–37^ and our earlier findings^33^. The designs covered the coding exons of the selected genes. The targeted regions (according to Human Assembly GRCh37/hg19) were captured using a SureSelect^XT^ Target Enrichment System and SureSelect^XT^ Low Input (Agilent), as described in the manufacturer's instructions. One hundred twenty-five genes were common to the two designs and are referred to as the common gene set (CGS) (Suppl. Table S7). Captured libraries were diluted to 8 pM for Illumina clustering, and paired-end sequencing was performed on MiSeq and NextSeq sequencers (Illumina Inc., San Diego, CA, USA).

Three independent analyses were carried out for each sample. We first used the tools available in the Variant Reporter instrument (Illumina). A second variant calling was done with VarScan 2.3.9 to detect the mutations of the files extracted from two sources: (1) BWA Enrichment of Illumina Base Space, and (2) the Burrows-Wheeler Aligner (BWA), Picard and Indel Realignment-Base Recalibration from the Genome Analysis Toolkit 3.8.1.0 (GATK).

Annotation was carried out with Annovar. All variants identified by the three complementary methods were visualized using an Integrative Genomics Viewer (Broad Institute and UC San Diego, San Diego, CA, USA). Data have been deposited in the Sequence Read Archive (SRA) (accession number PRJNA648645).

After annotation, the variants were subjected to additional, more stringent, and quality- and relevance-based filtering applying the following criteria: quality read depth of bases ≥ 50; depth of variant-supporting bases ≥ 5; localization (exonic, UTRs and splice site); variant effect (non-synonymous); variant allele frequency ≥ 5% and not listed as a single nucleotide polymorphism, or listed but with a MAF < 0.01% (The Exome Aggregation Consortium, 1000 Genomes Project of the International Genome Sample Resource (IGSR), Single Nucleotide Polymorphism Database (dbSNP) v138 of the National Center for Biotechnology Information (NCBI)). The median coverage was 764x (89–1908) (Suppl. Table S8).

### Tissue microarray construction, immunohistochemistry and in situ hybridization analyses

Thirty-one DLBCL samples for which sufficient biopsy material was available were included in a tissue microarray (TMA). Known positive and negative controls (one tonsil, one lymphadenopathy, and a known triple-hit DLBLC case) were also included. A tissue arrayer (Beecher Instruments, Silver Spring, MD, USA) was used for TMA construction. Hematoxylin and eosin-stained sections from each biopsy were used to define the appropriate areas, and two representative cores (1 mm diameter) were used for the TMA.

TMA sections were stained with antibodies against CD10 (clone 56C6), BCL-6 (PG-B6p), MUM1 (MUM1p), BCL-2 (124), and p53 (D07), all of which were mouse monoclonal antibodies, obtained from Agilent-Dako (Santa Clara, CA, USA), and MYC (Y69, rabbit polyclonal; Agilent-Dako). The COO based on IHC was established by the Hans algorithm^38^. EBER in situ hybridization (EBER1 DNA probe, Roche-Ventana; Basel, Switzerland) was carried out to detect EBV.

Fluorescence in situ hybridization (FISH) was performed following the routine protocol at the Pathology Department of our Institution. ZytoLight Dual Color Break Apart probes (ZytoVision GmbH, Bremerhaven, Germany) were used to analyze *BCL2* (18q21 region), *BCL6* (3q27), and *C-MYC* (8q24).

IHC and FISH data from the samples not included in the TMA were collected from the original pathological reports.

### Nanostring LST nCOUNTER gene-expression assay

We used the NanoString LST assay performed with the nCounter Dx Analysis System (NanoString Technologies, Seattle, WA, USA) to determine COO. Total RNA from 54 FFPET sections from diagnostic samples was isolated using a truXTRAC FFPE total NA kit (Covaris) following the manufacturer's instructions. RNA quality and quantity were assessed with an RNA 6000 Nano kit (Agilent) using the Agilent 2100 Bioanalyzer System (Agilent).

The previously reported protocol for the NanoString LST gene expression assay was used^39,40^. Briefly, the probes corresponding to the 20 LST genes^41,42^ and based on the Lymph2Cx gene expression assay were hybridized in a multiplexed reaction to 400 ng of the total RNA for 16 h at 65 °C. Automated removal of excess probe and immobilization of probe–transcript complexes on a streptavidin-coated cartridge were carried out in the nCounter Prep Station, and raw gene expression counts were collected on the Digital Analyzer. Data were analyzed using nSolver 4.0 software (NanoString Technologies) for COO classification.

### Genetic classification

We performed a simplified genetic classification based on those proposed by Schmitz-Wright^12,13^ and Lacy^15^ and their research teams. We used the alterations of specific genes to develop a two-step classification method and classified the samples as N1^2-S^, BN2^2-S^, EZB^2-S^, MCD^2-S^, or ST2^2-S^. The A53 LymphGen subtype was not included due to the lack of copy number variation data in our series and most published series. The genes from the Schmitz-Wright^12,13^ and Lacy^15^ studies were selected by calculating their power to classify determined by the Fisher's exact test, as featured in these studies (Suppl. Table S9). Several tests were then carried out to select the best combination of genes, taking into account the sensitivity and specificity in the PdH and HMRN cohorts according to the LymphGen and Lacy classification (AIC cluster). In the first step, at least one of the top genes should be mutated: *NOTCH1,* for N1^2-S^ (*NOTCH1* mutated samples were classified as N1^2-S^, regardless the presence of other alterations); *MYD88*, *CD79B*, and *PIM1* for MCD^2-S^; *BCL6* translocation, *NOTCH2*, *BCL10*, and *TNFAIP3* mutations for BN2^2-S^; *BCL2*, *EZH2*, and *CREBBP* for EZB^2-S^; and *SGK1*, *TET2*, and *SOCS1* for ST2^2-S^. Samples with the same score for two or more subtypes, or samples with no mutations, were classified in the second step, in which we added the following genes for each subtype: *PRMD1*, *BTG1*, *PIM2*, and *CD58* for MCD^2-S^; *UBE2A*, *CD70*, *CCND3,* and *DTX1* for BN2^2-S^; *TNFRSF14*, *KMT2D*, *IRF8*, and *EP300* for EZB^2-S^, and *STAT3* for ST2^2-S^. In this second step, at least two genes from any of the genes that define each subtype should be mutated to assign the sample to the corresponding subtype (Fig. S5 and Suppl. Table S9).

We selected the HMRN cohort^15^ as an external validation cohort for the two-step method, restricting the analysis to patients treated with R-CHOP (n = 580).

### Sensitivity and specificity

The sensitivity and specificity of each subtype defined by the two-step method were determined using the LymphGen and AIC classifiers as references in the PdH and HMRN cohorts. Sensitivity was calculated as the percentage of true positives relative to the reference classifier:

$$Sensitivity = \frac{True \,positives}{{True \,positives + False \,negatives}}.$$

Specificity was calculated as the percentage of true negatives relative to the reference classifier:

$$Specificity = \frac{True \,negatives}{{True \,negatives + False \,positives}}.$$

### Statistical analysis

Statistical analyses were performed using R 3.6.1 (https://www.R-project.org, R Foundation for Statistical Computing, Vienna, Austria). Associations between factors were assessed with Fisher's exact test and the predictive value of the selected variants by Cox proportional-hazards models, adjusted using the Benjamini–Hochberg correction method.

OS was calculated as a binomial factor for survival or non-survival of each sample. Progression-free survival (PFS) was calculated as a binomial factor, assigning the event to the treated patients who did not respond to treatment or who relapsed. We performed univariate and multivariate logistic regression analyses of genes and pathways mutated in at least four samples to establish each variable's predictive value. Hazard ratios (HRs) with 95% confidence intervals were estimated for each variable. The survival function of genetic groups, as measured by OS and time to relapse (PFS), was also calculated by the Kaplan–Meier method.

## Supplementary information

Supplementary Information

Supplementary Information

## Data Availability

Accession codes: SRA (PRJNA648645). The R code for the two-step classifier can be downloaded from https://github.com/Lymphoma-IDIPHISA/Two-step-classifier.
