# Supplementary material for: Proposal and validation of a method to classify genetic subtypes of diffuse large B cell lymphoma
Source: Sci Rep. 2021 Jan 21;11:1886. doi: 10.1038/s41598-020-80376-0 (PMC7820010; doi:10.1038/s41598-020-80376-0)
Supplement: Supplementary file 2 — Supplementary Information [file 41598_2020_80376_MOESM2_ESM.pdf]

Proposal and validation of a method to classify genetic subtypes of diffuse large B cell lymphoma

Lucía Pedrosa[1,2,+], Ismael Fernández-Miranda[1,2,+], David Pérez-Callejo[3,4,+], Cristina Quero[5], Marta Rodríguez[6,7], Paloma Martín-Acosta[7,8], Sagrario Gómez[1], Julia González-Rincón[1,7], Adrián Santos[8], Carlos Tarin[9,10], Juan F. García[7,11], Francisco R. García-Arroyo[12], Antonio Rueda[13], Francisca I. Camacho[14], Mónica García-Cosío[15], Ana Heredero[1], Marta Llanos[16], Manuela Mollejo[7,17], Miguel Piris-Villaespesa[18], José Gómez-Codina[19], Natalia Yanguas-Casás[1], Antonio Sánchez[3], Miguel A. Piris[6,7], Mariano Provencio[3], Margarita Sánchez-Beato[1,7*]

[1]Lymphoma Research Group, Medical Oncology Department, Instituto de Investigación Sanitaria Puerta de Hierro-Segovia de Arana, Madrid, Spain.

[2]PhD Program in Molecular Biosciences, Doctoral School, Universidad Autónoma de Madrid, Spain.

[3]Medical Oncology Department, Hospital Universitario Puerta de Hierro, Madrid, Spain.

[4]PhD Program in Medicine and Surgery, Doctoral School, Universidad Autónoma de Madrid, Spain.

[5]Medical Oncology Department, Hospital Universitario Virgen de la Victoria, Malaga, Spain.

[6]Pathology Department, Hospital Fundación Jiménez Díaz, Madrid, Spain.

[7]Centro de Investigación Biomédica en Red de Cáncer (CIBERONC), Madrid, Spain.

[8]Molecular Pathology Laboratory, Instituto de Investigación Sanitaria Puerta de Hierro-Segovia de Arana, Madrid, Spain.

[9]Bioinformatics Unit, Instituto de Investigación Sanitaria Puerta de Hierro-Segovia de Arana, Madrid, Spain.

[10]Basic Medical Sciences, Faculty of Medicine, Universidad CEU San Pablo, Madrid, Spain.

[11]Pathology Department, MD Anderson Cancer Center, Madrid, Spain.

[12]Medical Oncology Department, Complejo Hospitalario de Pontevedra, Pontevedra, Spain.

[13]Medical Oncology Department, Hospitales Universitarios Regional y Virgen de la Victoria, IBIMA, Malaga, Spain.

[14]Pathology Department, Hospital Universitario de Getafe, Madrid, Spain.

[15]Pathology Department, Hospital Universitario Ramón y Cajal, Madrid, Spain.

[16]Medical Oncology Department, Hospital Universitario de Canarias, Tenerife, Spain.

[17]Pathology Department, Hospital Virgen de la Salud, Toledo, Spain.

[18]Haematology Department, Hospital Universitario Ramón y Cajal, Madrid, Spain.

[19]Medical Oncology Department, Hospital Universitari i Politècnic La Fe, Valencia, Spain.

[+]L. Pedrosa, I. Fernández-Miranda and D. Pérez-Callejo contributed equally to this work.

***Corresponding Author:**

Margarita Sánchez-Beato (PhD), E-mail: msbeato@idiphim.org

Instituto de Investigación Sanitaria Puerta de Hierro-Segovia de Arana,

Calle Joaquín Rodrigo 2, Majadahonda, 28222 Madrid, Spain.

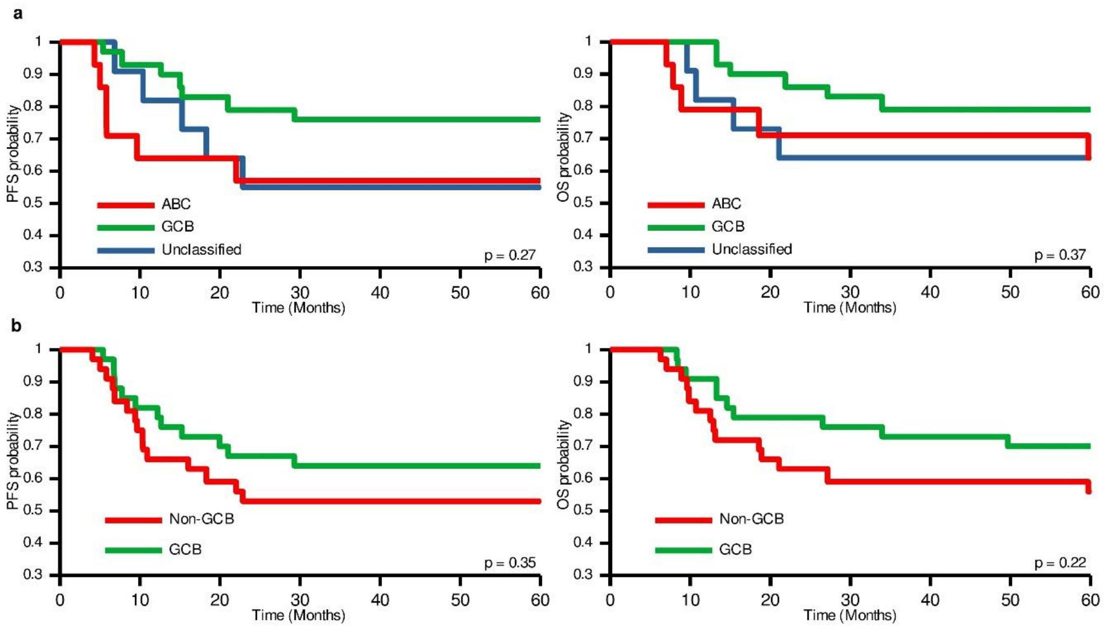

Figure S1. Progression-free survival (PFS) and overall survival (OS) Kaplan-Meier analysis of cell-of-origin (COO) classification in the PdH cohort. A) Lymph2Cx classification; **B)** Hans classification. ABC: activated B-cell; GCB: germinal center B-cell.

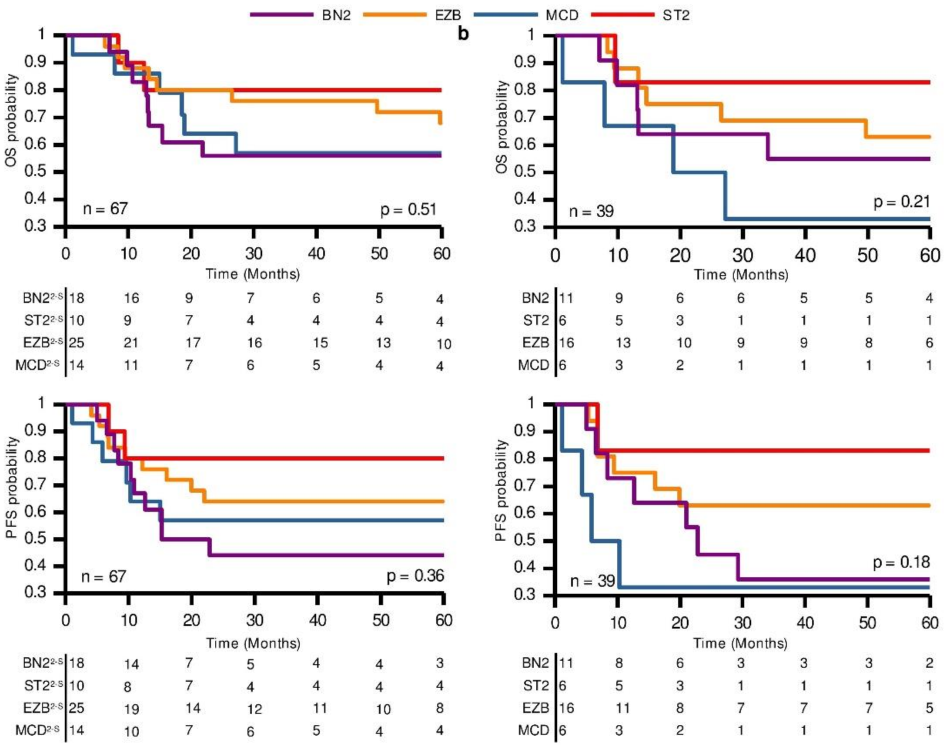

Figure S2. Genetic subtypes from two-step and LymphGen methods in the PdH cohort. Kaplan–Meier analysis of **A)** two-step and **B)** LymphGen genetic subtypes for overall survival (OS) and progression-free survival (PFS).

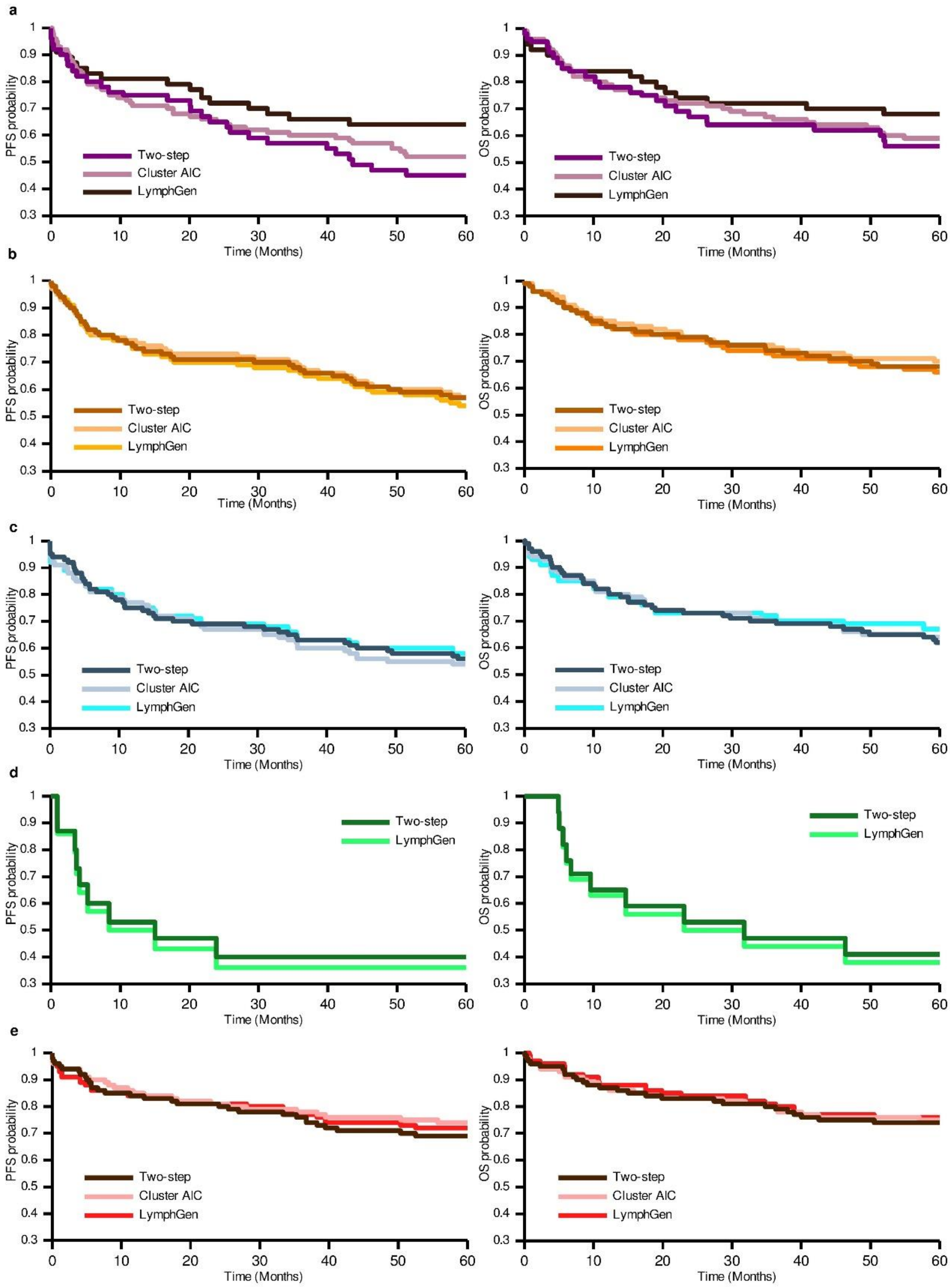

Figure S3. Comparison between the classifiers. Progression-free survival (PFS) and overall survival (OS) Kaplan-Meier analysis of **A)** $BN2^{2\text{-}S}$/BN2/NOTCH2, **B)** $EZB^{2\text{-}S}$/EZB/BCL2, **C)** $MCD^{2\text{-}S}$/MCD/MYD88, **D)** $N1^{2\text{-}S}$/N1 and **E)** $ST2^{2\text{-}S}$/ST2/SGK1-TET2-SOCS1 in the HMRN cohort.

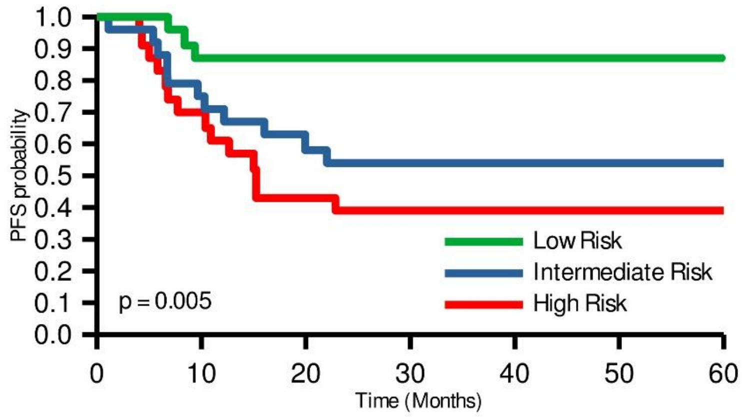

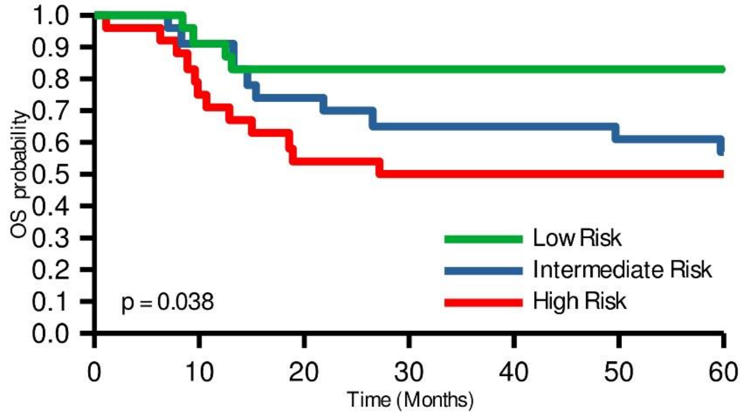

Figure S4. Combinational model of IPI and genetic subtypes from two-step method in the PdH cohort. Kaplan-Meier analysis of risk groups created by the Cox proportional-hazards multivariate model with IPI and genetic subtypes for progression-free survival (PFS) and overall survival (OS) status in the HMRN cohort and validated in the PdH cohort.

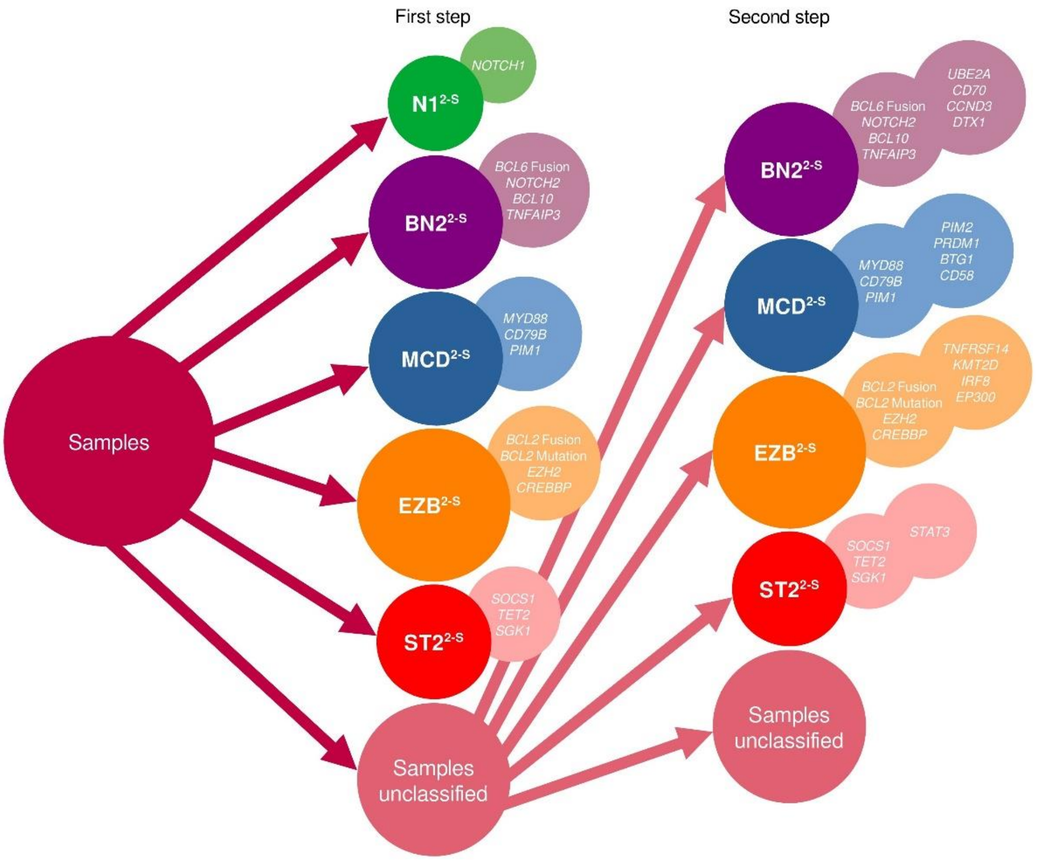

Figure S5. Schematic representation of the two-step classifier for assigning subtypes to a sample.
